# The near-symmetry of protein oligomers: NMR-derived structures

**DOI:** 10.1038/s41598-020-65097-8

**Published:** 2020-05-20

**Authors:** Maayan Bonjack, David Avnir

**Affiliations:** Institute of Chemistry, The Hebrew University of Jerusalem, Jerusalem, 9190401 Israel

**Keywords:** Proteins, Biochemistry, Structural biology, NMR spectroscopy

## Abstract

The majority of oligomeric proteins form clusters which have rotational or dihedral symmetry. Despite the many advantages of symmetric packing, protein oligomers are only *nearly* symmetric, and the origin of this phenomenon is still in need to be fully explored. Here we apply near-symmetry analyses by the Continuous Symmetry Measures methodology of protein homomers to their natural state, namely their structures in solution. NMR-derived structural data serves us for that purpose. We find that symmetry deviations of proteins are by far higher in solution, compared to the crystalline state; that much of the symmetry distortion is due to amino acids along the interface between the subunits; that the distortions are mainly due to hydrophilic amino acids; and that distortive oligomerization processes such as the swap-domain mechanism can be identified by the symmetry analysis. Most of the analyses were carried out on distorted *C*_*2*_**-**symmetry dimers, but *C*_*3*_ and *D*_*2*_ cases were analyzed as well. Our NMR analysis supports the idea that the crystallographic B-factor represents non-classical crystals, in which different conformers pack in the crystal, perhaps from the conformers which the NMR analysis provides.

## Introduction

The majority of proteins which form oligomers are organized in quaternary structures which are symmetric at least to some degree^1–3^. The most prevalent symmetry point-groups of these oligomers are the cyclic ***C***_***n***_ and the dihedral ***D***_***n***_ symmetries. The symmetric assembly of the oligomeric subunits leads to various advantages over asymmetric structure or monomeric form. These include coding efficiency and reduction of synthetic errors, cooperative regulation, increase in stability and formation of lower-energy structures, and minimization of excessive aggregation^2–7^. Interestingly, despite these many functional advantages of the symmetric packing, the vast majority of protein oligomers are only *nearly* symmetric^2,4–6,8–11^, that is, although many oligomeric proteins are classified as ‘symmetric’, there are conformational differences between the sequence-identical subunits^2,11^. Understanding the origin and role of the asymmetry, is critical for understanding protein functions. In a recent review, Goodsell highlighted the issues around the near symmetry of biological functions in general, and proteins in particular, and pointed to evolutionary processes as lead to symmetry at some degree^1^. The distortion from perfect symmetry of proteins is related to several issues, among them are functionality of the protein, thermodynamic considerations, such as enthalpy, entropy and the constant motion of the protein, the asymmetric environment that surrounds the protein, the mechanism of oligomerization, and even experimental conditions^1,9,11–14^. Symmetric proteins and their imperfection, have been an ongoing research and some recent studies include global motion patterns of symmetric proteins^15^, how symmetry can compound the effect of point mutations and trigger uncontrolled self-assembly into high-order structures^16^, and how symmetric protein can serve as starting points for diverse applications in medicine, biomaterials, and synthetic biology^12^.

Our interest in the phenomenon of imperfect-symmetry of proteins has focused on several points of view: Developing computational tools for the quantification of the symmetry deviations on whole-protein level^17^ and on fragments of it^13^; identifying the specific locations in the folded structure which carry most of the burden of pushing away the oligomer from ideal symmetry^13,14^; and identifying mechanisms that lead to that near-symmetry^14^. These studies have been based on the Continuous Symmetry Measures (CSM) methodology (explained below), which allows a quantitative treatment of that structural property. One of the main findings of that analysis identified the hydrophilic amino acids located near the symmetry-axis of the oligomer and in between its subunits as major contributors to the symmetry deviations. This occurs by adopting different conformers in the symmetry-related locations in the protein structure, using in a non-symmetric way, hydrogen and ionic bonds to minimize the enthalpy of the local amino-acid interactions^13^. Also, Ferreiro *et al*.^7^ pointed the interfaces in oligomeric proteins as reducing the interaction frustration thus leading to lower symmetry. Another main finding is related to the step-wise domain-swapping dimerization mechanism, identifying the hinge regions as the main locations responsible for the symmetry deviation in this mechanism^14^.

All of our protein symmetry analyses and all of the conclusions drawn from them so far, were based on x-ray crystallographic structural data. However, another powerful tool in structural protein studies is NMR spectroscopy. The two methods are inherently very different: While x-ray analysis provides information on the protein in its crystallized form, NMR relates to the more natural state of proteins, that is, to their conformational structures in water. Not only that the environments in these two cases are very different, but so are also the methodologies of the structure elucidation. We recall that NMR structural analysis is based on various types of 1D and 2D spectra, which provide indirect measures of restrictions on neighboring atoms, on dihedral-angles, on proton-proton distances, on hydrogen bonds, on bond lengths and more^18,19^. This data is translated to a set of co-existing conformers, typically 10–30 structures. The NMR-derived structures are not an image in the sense that x-ray structures are: in x-ray crystallography, 3D density map of electron densities are produced, representing the mean positions of the atoms, typically for a single conformer, while NMR analysis generates computationally derives 3D molecular models.

It is therefore of interest to explore the proteins near-symmetry phenomenon in solution in order (a) to see to what extent do conclusions drawn from crystallography still hold in solution; (b) to search for new insights on the origin of symmetry deviations that can be drawn from this type of analysis; and (c) to identify the differences between the structure in the crystal and the structure in solution from the symmetry perspective. Most of the manuscript focuses on the most common protein symmetry – the dimeric rotational ***C***_***2***_ symmetry – but we carry also comparative analyses of higher symmetries (***C***_***3***_, ***D***_***2***_). Some of our main findings detailed below are that the symmetry deviations as evaluated by the CSM analysis for homomers in solution, are by far higher – that is, more distorted - in solution, compared to the CSM values obtained in the crystalline state; that, as in the crystalline state, much of the symmetry distortion is concentrated in amino acids along the near-symmetry axis; and, that despite the aqueous environment, this distortion is mainly due to interactions between hydrophilic amino acids along the border between the homomer units, resulting mainly in asymmetric hydrogen bonds which link the subunits; we show, using NMR data, the importance of domain swapping mechanism in inducing asymmetry in homomers; and we show that the higher symmetries - ***C***_***3***_, ***D***_***2***_ – require more case-focused analyses, which depend on the specific geometries that carry these symmetries. Last but not least, a proposition which may emerge from our NMR data analysis is that the crystal of a protein is in fact not the classical case of having the exact same conformer repeating itself in an ideal translational symmetry, but that instead, the translational movement may lead to different conformers along that path, perhaps from the family of the conformers which the NMR analysis identifies. We are not the first to come up with the idea that protein crystals are non-classical from that point of view: For instance, Woldeyes *et al*.^20^, proposed that many protein conformers are populated in a single crystal, and that this conformational heterogeneity is averaged in time and space in x-ray crystallography datasets; our study supports this notion.

## Methods

### The continuous symmetry measure

The literature on proteins symmetry uses qualitative phrases, such as “nearly symmetric”, “quasi symmetric”, “far from being symmetric”, “perfectly symmetric”, and so on. The Continuous Symmetry Measure (CSM)^21,22^ is a method for quantifying such phrases by assigning a *degree* of a given symmetry. According to the CSM approach, the symmetry measure of a ***G***-symmetry point group content of an object is a function of the distance between the original structure and a searched ***G***-symmetric reference structure, of the same atoms and connectivity and which is the closest to the original distorted structure. This minimal distance of the object’s vertices from the investigated ***G***-symmetry defines the measure *S(****G***):

1 $$S({\boldsymbol{G}})=\frac{100}{{d}^{2}}\mathop{\sum }\limits_{i=1}^{N}{|{\bar{Q}}_{i}-{\bar{Q}}_{i}^{sym}|}^{2},$$

where ${\bar{Q}}_{i}$ are the coordinates of the i^th^ atom of the original studied molecule, ${\bar{Q}}_{i}^{sym}$ are the coordinates of the i^th^ atom of the nearest structure which has the investigated symmetry, the denominator is the root mean square size normalization factor of the originally centered structure ($d=\sqrt{\mathop{\sum }\limits_{i=1}^{N}{|{\bar{Q}}_{i}|}^{2}}$)), and *N* is the number of analyzed atoms in the structure (see full details in ref.’s^13,23^). It should be emphasized that this measure is inherently different form symmetry analyses which are based on rmsd calculations of the degree of similarity, as the rmsd analysis does not evaluate how symmetric are the different parts in the structure, but only evaluates how similar they are. The range of the CSM values is $0\le S({\boldsymbol{G}})\le 1$ and it is expanded by a factor of 100 for convenience ($0\le S({\boldsymbol{G}})\le 100)$. For an object which has perfect ***G***-symmetry the $S({\boldsymbol{G}})=0$, since the minimal distance between ${\bar{Q}}_{i}$ and ${\bar{Q}}_{i}^{sym}$ is zero, and as the object distorts from the perfect symmetry, *S(****G***) increases. *S(****G***) is a special distance function in that the nearest symmetrical structure ${\bar{Q}}_{i}^{sym}$ is usually not known *a-priori*, and is determined by minimization protocols described in detail in previous publications^21,24–26^. Online CSM calculators are available at: http://www.csm.huji.ac.il and http://csm.ouproj.org.il. The CSM measure is a global parameter, and thus allows the comparison of various structures and various symmetries on the same scale. Numerous applications of the CSM methodology all across chemistry have been reported and few examples are collected in the cited references^27–30^, including biochemistry^14,31,32^ and physics^33–37^.

### Types of symmetry analysis

In previous studies^13,14^ we have introduced specific CSM computational tools for the evaluation of the symmetry content, *S(****G****)*, of proteins. Four variations of which are relevant for this report: The “all-atoms symmetry analysis”, “local symmetry analysis”, “symmetry maps analysis” and “symmetry spectrum analysis”:

The ***all-atoms analysis*** includes the whole structure of the protein, that is, the backbone atoms and the residues atoms which, in NMR structures – unlike most of the crystallographic derived structures – include the hydrogen atoms. This measure enables the user to have a general sense of the symmetry of the protein as a whole, and finds the location of the symmetry axis which provides the minimal CSM value.

The ***local symmetry analysis*** is a high resolution tool which focuses on symmetry relations between specific amino-acids (pairs in the case of ***C***_**2**_-symmetry, triplets in the case of ***C***_***3***_-symmetry, and so on) in the protein structure. A CSM calculation is carried out on each set (pair, in the case of ***C***_**2**_-symmetry) of symmetry-related amino acids within the oligomer, one amino-acid from each monomer. Each such calculation provides a local CSM value and this reveals, on one hand, which pairs of amino-acids are the most distorted ones in the structure, carrying the burden of the symmetry deviation of the whole, and on the other hand, which are barely deviating from perfect symmetry. Detailed examples below will clarify it further.

The ***symmetry maps*** analysis provides a telltale visualization of the local symmetry deviations, and is based on the collection of all of the local CSM values, displayed with a color-code. A graphical decision is made as to the color code of amino acids that refers to very-high, high, medium, low and very-low CSM values.

The ***symmetry spectrum***, as was introduced in ref. ^14^, is a plot of the CSM of the protein, which is constructed as follows (explained for *S(****C***_**2**_): A segment of *h* amino-acids is selected (*h* is defined as the size of analysis ruler). Then, starting with the 1^st^ amino-acid in the polypeptide chain of the monomer, the *S(****C***_***2***_*)* value of the first ***C***_***2***_-symmetry-related segments (1^st^-*h*^th^ amino-acids segments- pair) is calculated, and a first CSM value is obtained. The ruler is moved then by a one amino-acid step to the second segment − 2^nd^-(*h* + 1)^th^ amino-acids - and a second CSM value is calculated. The procedure is repeated one amino-acid after the other with the “running ruler” until (and including) the final segment of length *h* is reached. A total of $N=n-h+1$ (where n is the number of amino-acids in the subunit) segments and their associated CSM values are obtained. A CSM spectrum is then plotted in which the CSM value (*S(****C***_***2***_*)*) of the i-th segment (y-axis) is presented as a function of the position, *n*_*i*_, of the first amino acid in that segment (x-axis). Segments of high symmetry distortion appear then as distinct peaks.

### The analyzed protein data

We focused mainly on dimers (with ***C***_***2***_ symmetry), but trimers and tetramers - with ***C***_***3***_*-* and ***D***_***2***_-symmetries, respectively - are included as well. The coordinates of 45 analyzed proteins [*Q*_*i*_ in Eq. (1)], listed throughout this paper, were obtained from the Protein Data Bank (PDB)^38^. Only structures with 20 submitted conformers or more were taken. Redundant entries with more than 30% identity were filtered out. Regarding the quality of the analyzed data, we followed the recommendations of Kwan *et al*.^19^ in selecting the structures analyzed below; that is, we chose structures which have a backbone rmsd <0.5 Å and a Ramachandran Plot quality >95%. A further filter was to use only structures with chain length of more than 50 well-defined amino-acids. We have ignored the coordinates of residues which are classified in the PDB file as ill-defined. We did not use any data which was derived by assuming symmetry - these structures are, by definition, fully symmetric^1,39^, that is, of S(G) = 0 value. For comparative purposes we also created average structures by averaging the coordinates of each atom from all conformers of the same protein.

**Table 1:** The ten most distorted amino acid residues in the three conformers of Fig. 1 and their local S(**C**_**2**_) symmetry distortion values.

Minimal energy residue number [S(*C*_*2*_)]	Least distorted residue number [S(*C*_*2*_)]	Most distorted residue number [S(*C*_*2*_)]
Lys11 [0.38]		
		His24 [0.80]
Gln35 [0.77]		Gln35 [0.46]
Leu38 [1.0]	Leu38 [1.8]	Leu38 [1.0]
Arg39 [0.58]	Arg39 [1.3]	Arg39 [4.8]
Arg40 [19]	Arg40 [0.16]	Arg40 [14]
Glu41 [2.1]		Glu41 [0.91]
Tyr43 [0.59]	Tyr43 [0.71]	Tyr43 [0.83]
	Asp44 [0.24]	Asp44 [0.37]
Phe47 [0.44]	Phe47 [0.37]	Phe47 [2.3]
Arg48 [0.41]	Arg48 [0.48]	Arg48 [0.86]
Asp49 [0.50]		
	Leu50 [0.19]	
	Cys51 [0.21]	
	Leu67 [0.20]

**Table 2:** The ***C***_***2***_-CSM values of the most and least distorted conformers of each protein and of the averaged structures with their B-factors.

PDB ID	Min CSM	Max CSM	Averaged structure CSM	B-factor(Average structure)
2MX9	0.0587	0.1627	0.0073	25.30
2MJA	0.0013	0.0531	0.0036	99.90
2MVW	0.2917	0.5554	0.0746	34.06
2MFZ	0.0209	0.1094	0.0116	12.74
2MGS	0.0380	0.1486	0.0022	9.70
2MAJ	0.0013	0.0088	0.0005	25.23
2LRM	0.2377	0.4938	0.0139	45.26
2LYJ	0.1140	0.1960	0.0064	20.39
2LTD	0.0944	0.3125	0.0195	19.76
2LJY	0.1336	1.4153	0.1329	88.64
2K9I	0.1822	0.4816	0.0246	28.98
2KJZ	0.0146	0.1485	0.0052	27.76
2K7I	0.1035	0.3582	0.0116	35.04
2K29	0.0825	0.4303	0.0153	22.62

See Supplementary Table S1 for more proteins.

## Results and Discussions

### Comparative C_2_-symmetry analysis of three conformers of the HPV16 E6 dimer

**Figure 1:**
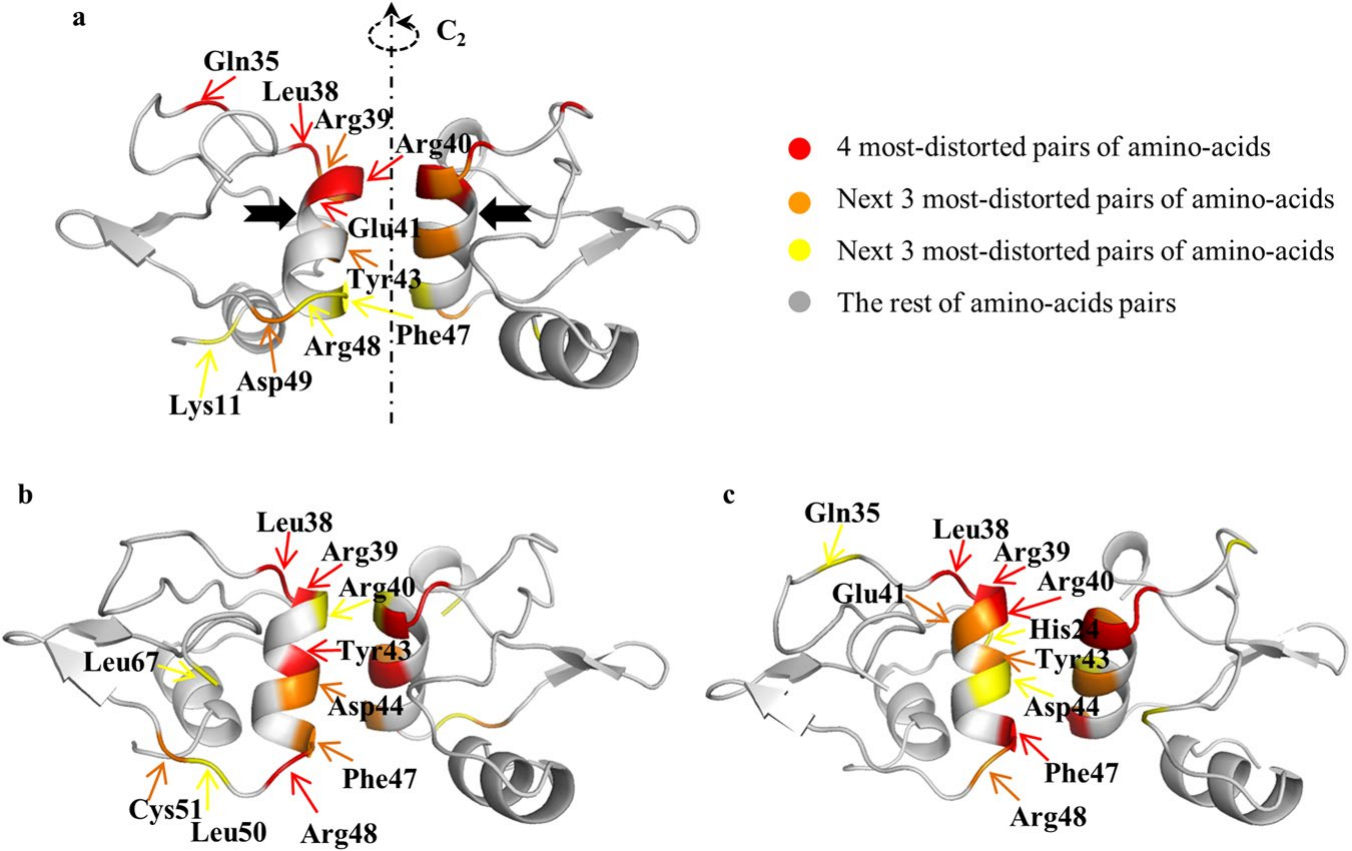
Symmetry maps of three NMR-derived conformers of the N-terminal domain dimer of HPV16 E6 (PDB 2LJY): (**a**) The conformer with the minimal energy. Here, red - the four most symmetry-distorted pairs (1.0 < S(***C***_***2***_) < 20); orange - the next three lower symmetry-distorted pairs (0.45 < S(***C***_***2***_) < 1.0); yellow - the next lower three most symmetry-distorted pairs (0.38 < S(***C***_***2***_) < 0.45); grey – the rest of the amino acids (S(***C***_***2***_) < 0.38). (**b**) The least ***C***_***2***_-symmetry distorted conformer – see Table 1 for details. (**c**) The most symmetry distorted conformer – see Table 1 for details. Note that because of the near ***C***_***2***_-symmetry, the left arm colors of the dimer are in the back of the right arm, and *vice-versa*. The black arrows shown in **(a**) indicate the locations of the quite symmetric Val42 pair (gray, S(***C***_***2***_) = 0.044) within an otherwise distorted zone – see text for explanation.

We begin with the details of the symmetry-distortion analysis and of the structural information that can be drawn from it, using an exemplary protein, and then generalize the findings to the full set of oligomers analyzed in this study. We selected for that purpose the N-terminal domain dimer of HPV16 E6, a viral oncoprotein which is an essential factor for cervical cancers induced by “high-risk” mucosal HPV (PDB 2LJY^40^). Zanier *et al*.^40^ have calculated 20 NMR-based conformers structures for that protein, and of those we begin with three special conformers – the one that is of minimal energy as assigned in the original report (en); the conformer that is the least distorted from ***C***_***2***_-symmetry (min); and the one that is the most distorted from this symmetry (max). The CSM values of these three conformers, using the ‘all-atoms symmetry analysis’ were found to be S(***C***_***2***_)_en_ = 1.39, S(***C***_***2***_)_min_ = 0.13, and S(***C***_***2***_)_max_ = 1.42, respectively. What do these numbers represent from the point of view of the symmetry distortions of specific amino acids symmetry-related pairs, one in each of the two monomeric units? For that purpose we examine now the symmetry maps of these conformers, shown in Fig. 1 and detailed in Table 1. These maps highlight, using the ‘local symmetry analysis’, the amino acid residues which contribute most to the overall CSM value and the regions where these distortive amino acids reside. The range of local S(***C***_***2***_) values presented in symmetry maps, the dissection of that range into smaller ranges, the colors code used to represent these CSM ranges, and the number of amino acid pairs to be analyzed, are all open to selection, dictated by what aspects of the distortion one wishes to analyze. Figure 1 shows typical convenient selection of these parameters, suitable for the discussion which follows. Here, the selection was to indicate in each such map the 10 most ***C***_***2***_-symmetry-distorted symmetry-matched pairs of amino-acids. For example, in the symmetry map sown in Fig. 1a, the 4 amino-acids pairs colored red are the most distorted in the conformer structure and are of CSM values range of 1.00–18.98, the orange color indicates the next 3 amino-acids pairs which in this specific conformer have CSM values of 0.50 < CSM < 1.00, and finally, 0.38 < CSM < 0.45 are the yellow pairs. All other amino-acids pairs of lesser distortion are colored grey. Another option of selection of the color code is to unify it for all compared maps, and this option is shown in Supplementary Fig. S1.

Here are some of the observations and conclusions which can be reached by observing the three maps (Fig. 1) and the data in Table 1: 1. The three maps share six pairs of distorted amino-acids out of the ten most distorted ones of each conformer (Leu38, Arg39, Arg40, Tyr43, Phe47, Arg48). 2. Most of the distorted pairs are located at the interface between the monomeric subunits; that is, the zone where the two components of the dimer interact with each other – the two alpha helixes in touch and the amino acids at their ends (Leu 48 and Arg 38) - are very active in inducing the symmetry distortions. The observation is that the mutual adjustment of the orientations of the functional moieties of the amino acids for minimal energy interaction, leads to the compromise of the symmetry. 3. The other distorted amino acid residues reside in irregular elements. We also calculated separately the symmetry distortion of rigid secondary structures (α-helixes and β-sheets) and of flexible ones, and found that their CSM values are comparable −1.49 and 1.27 − respectively, that is, perhaps unexpectedly, rigidity is no safeguard from symmetry distortion. 4. The importance of the touching zone between the monomeric units is nicely evident by noticing that in the sequence of distorted amino acids 38–43 (Table 1, lowest energy conformer and most distorted conformer), the pair of Val 42 keeps its relatively high symmetry despite its distortive neighborhood (Fig. 1a): The local CSM value of Val 42 is S(***C***_***2***_) = 0.044, whereas its closest neighbors, Glu41 and Tyr43 have higher local CSM values of 2.1 and 0.59, respectively. This is so, not only because it is a hydrophobic amino acid but also because within the alpha-helix it is located away from the touching zone. We have also calculated for conformer a (Fig. 1a) what is the contribution of the interface residues to the overall symmetry distortion of the protein and found the CSM to be 0.44 out of a total value of 1.39; that is, a sixth of the amino acids of the protein are in the interface (11 out of 67 pairs) and contribute a third (32%) to the overall distortion – see details in Supplementary Fig. S2. 5. In Table 1 it is seen that the energetically stable conformer and the most distorted one share common features, more than with the least distorted one. This however, turned out not to be a general feature when the full library of proteins is analyzed. An additional option of local symmetry analysis is to use the nearest C_2_ axis obtained for the whole protein dimer, and the results are collected in Supplementary Table S2. It is seen that although the local CSM value of each pair might differ, almost the exact same ten most distorted amino acid residues are obtained.

**Figure 2:**
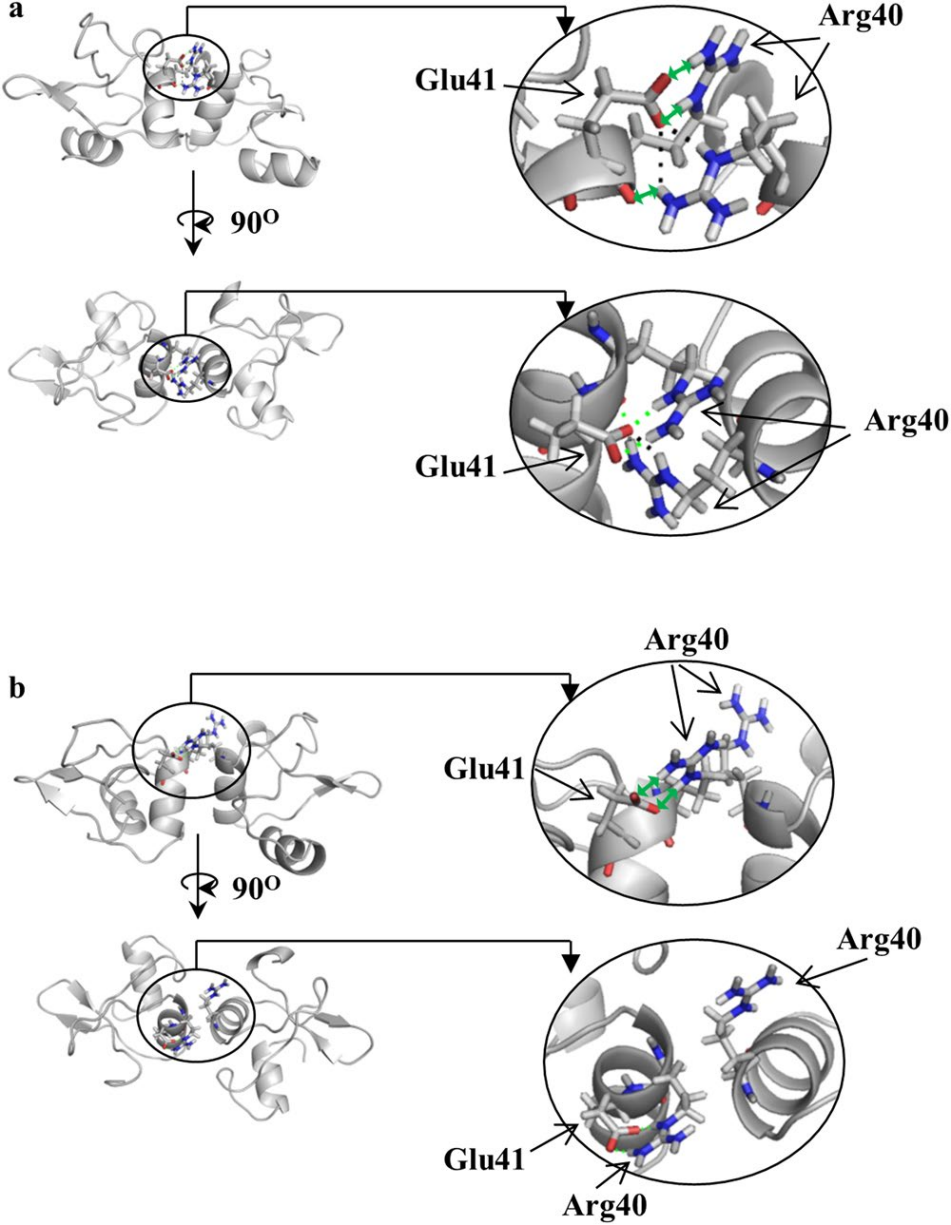
Zoom-in on Arg40 of N-terminal domain dimer of HPV16 E6 in the dimer interacting zone (red - oxygen, blue - nitrogen). (**a**) Left: The minimal energy conformer (conformer (a) in Fig. 1). Right: Shown are the distortive hydrogen bonds between Arg40 from one subunit and Glu41 from other subunit (black dashed line) and the distortive hydrogen bonds between the two Arg40 and between Arg40 and Glu41 from the same subunit (two-way green arrows). (**b**) The least ***C***_***2***_ -symmetry distorted conformer (conformer (**b**) in Fig. 1): Only intra-helical hydrogen bonds are formed between the two Arg40 (green dashed line). See text for detailed explanation.

Next, let us focus on Arg40 which is the most distorted pair of amino-acids in two conformers - the minimal energy conformer, and the most distorted one: extreme S(***C***_***2***_) values of 19 and 14 are recorded for these two, respectively. This amino-acid is located, as noted above, in the interacting zone of the dimer monomeric units, within the Arg39-Arg40-Glu41 colored region in the symmetry maps. A closer look of this amino acid pair mutual orientation is provided in Fig. 2: Being at an alpha helix capping position, Arg40 lacks intra-helical hydrogen bonds, but instead is stabilized by hydrogen bonds with the counter monomeric unit (Fig. 2a). This is probably general, because the alpha helix capping motif is found at or near the ends of helices which therefore necessarily lacks intra-helical hydrogen bonds and often uses alternative hydrogen bond partners for capping. Specifically the guanidino moiety of Arg40 from one monomeric unit forms a hydrogen bond with the carbonyl group of Glu41 in the second monomeric unit (marked as black dashed line in Fig. 2). In order to enable this hydrogen bond, the symmetry-related guanidino moiety of Arg40 from the second subunit must adopt a completely different conformation. The resulting mutual orientation of the two Arg40, is very far from being ***C***_***2***_-symmetric. If symmetry would have been preserved, then the formation of this stabilizing hydrogen bond would have been impossible. In fact, this distortion which induces the non-equivalence of the Arg40 pair also makes possible the formation of more hydrogen bonds – see the green dashed line between Arg40 from both subunits and between Arg40 and Glu41 in the same subunit. This special situation is highlighted by comparison with the Arg40 pair in the least symmetry distorted conformer (Fig. 1b). In this conformer the S(***C***_***2***_) value is ten-fold lower (0.16, Table 1), but still one of the ten most-distorted pairs of amino acids) compared with the two other conformers: the two Arg40 adopt relatively similar conformations (yet still not fully symmetric), which prohibit hydrogen bonding between the guanidino end of Arg40 from one unit and the carbonyl group of Glu41 from the other subunit (Fig. 2b). Only intra-hydrogen bonds between the residues of Arg40 with the residue of Glu41 of the same subunits are formed (marked as green dashed line in Fig. 2b). It is interesting to ask if the higher overall S(***C***_***2***_) values of these two conformers (S(***C***_***2***_)_en_ = 1.39 and S(***C***_***2***_)_max_ = 1.42) compared with the most symmetric conformer (S(***C***_***2***_)_min_ = 0.13) are due solely to the highly distortive Arg40 in these two conformers: When Arg40 is removed from the calculation, the value of S(***C***_***2***_)_en_ drops to 1.1, still by far more distorted than the most symmetric conformer. That is, the whole library of the local CSM values of the amino acids is important in determining the overall asymmetry of the oligomers.

As we have shown, the higher symmetry distortions mostly occur at the binding regions. Having analyzed in detail three of the 20 NMR-derived conformers of the N-terminal domain dimer of HPV16 E6, we move now to the full picture of all 20 conformers of this ptrotein.

### C_2_-symmetry analysis of the full set of conformers of the HPV16 E6 dimer

**Figure 3:**
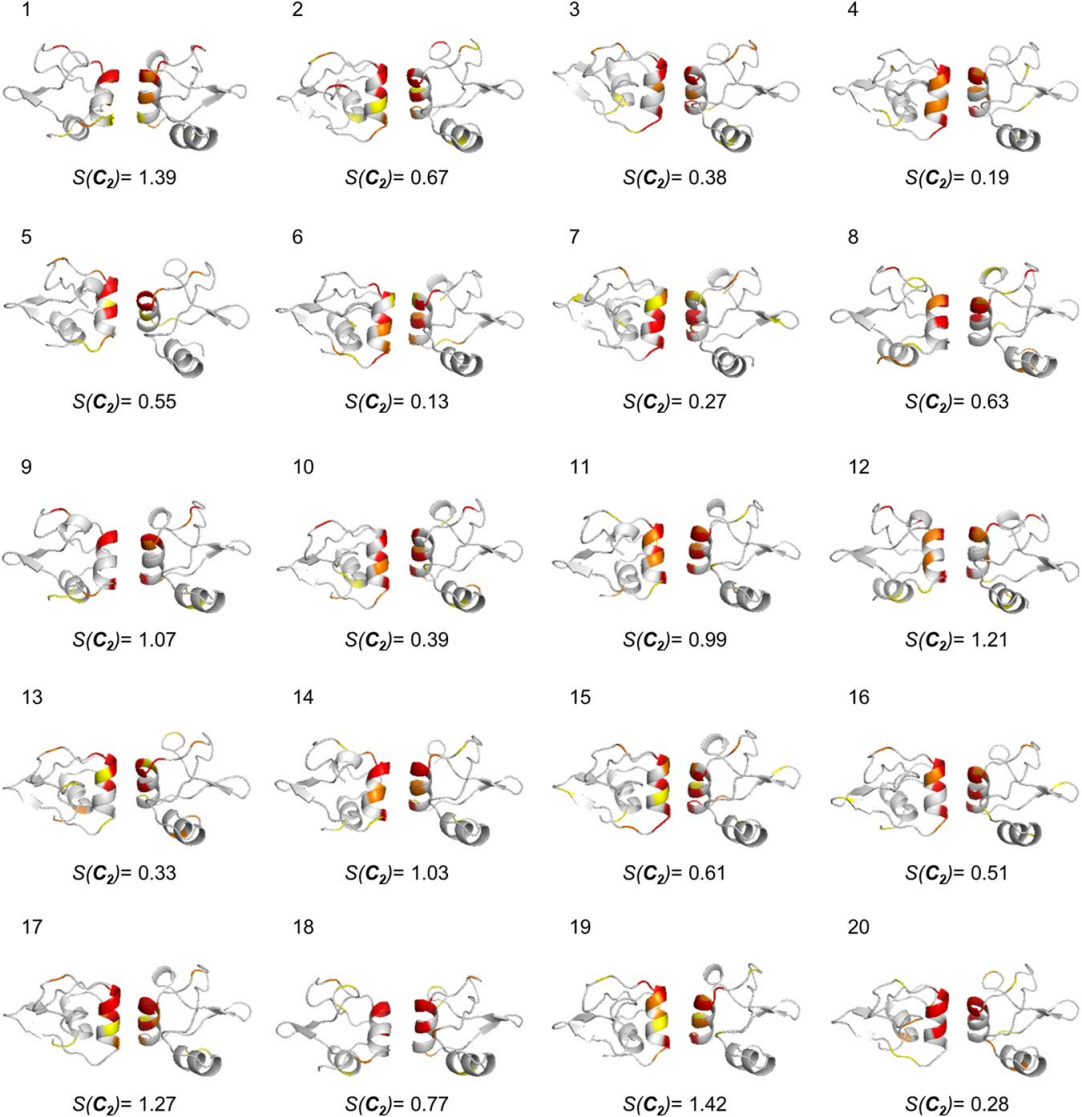
Symmetry maps of the 20 conformers of the N-terminal domain dimer of HPV16 E6 and their CSM values. The 10 most ***C***_***2***_-symmetry distorted symmetry-matched pairs of amino-acids are indicated in each conformer. The three conformers that were analyzed in Fig. 1 are conformer 1 (minimal energy), conformer 6 (the least distorted conformer) and conformer 19 (the most distorted). Color-code as in Fig. 1.

Having analyzed in detail three of the 20 NMR-derived conformers of the N-terminal domain dimer of HPV16 E6, we move now to the full picture of all 20 conformers of this protein. The symmetry maps of the whole family of conformers are collected in Fig. 3, along with the CSM values of the whole dimeric protein conformers, and the distribution of these values is provided in Supplementary Fig. S3. It is seen that the observation presented above is general, namely that the zone where the two components of the oligomer interact with each other is where much of the symmetry distortion takes place. Moreover, Arg40 which was discussed in detail above, is one of the 10-most ***C***_***2***_-distorted amino acids pairs in all of the 20 protein’s conformers with no exception.

We recall that the detection of the intercation zone of the monomeric units as the most responsible for the symmetry distortion was made also for the analysis of the x-ray crystallographic data^13^. This similarity is not trivial because the protein environments in the two types of analysis are very different: neighboring protein molecules in the crystalline state vs dissolution in water. There is, however, a major difference in the *magnitude* of the distortion, which is much higher in solution for *all* conformers (even the most symmetric), as revealed from the NMR data, ranging in Fig. 3 between S(***C***_***2***_) = 0.13 and 1.42; this is typically at least 10 fold higher compared with distortions revealed from crystallographic data. This conclusion does not change even if one takes into account that while NMR derived structures include the hydrogens of the proteins coordinates, most of the crystallographic data lacks it: As shown in Supplementary Table S3, which compares the CSM values of the 20 NMR conformers with and without the hydrogens, the values are similar, and high in both cases. This similarity was observed for other proteins analyzed in this study, and is a general trend. We return with an interpretation for the x-ray vs NMR differences, below.

### C_2_ - symmetry analysis of the full set of dimeric proteins

**Figure 4:**
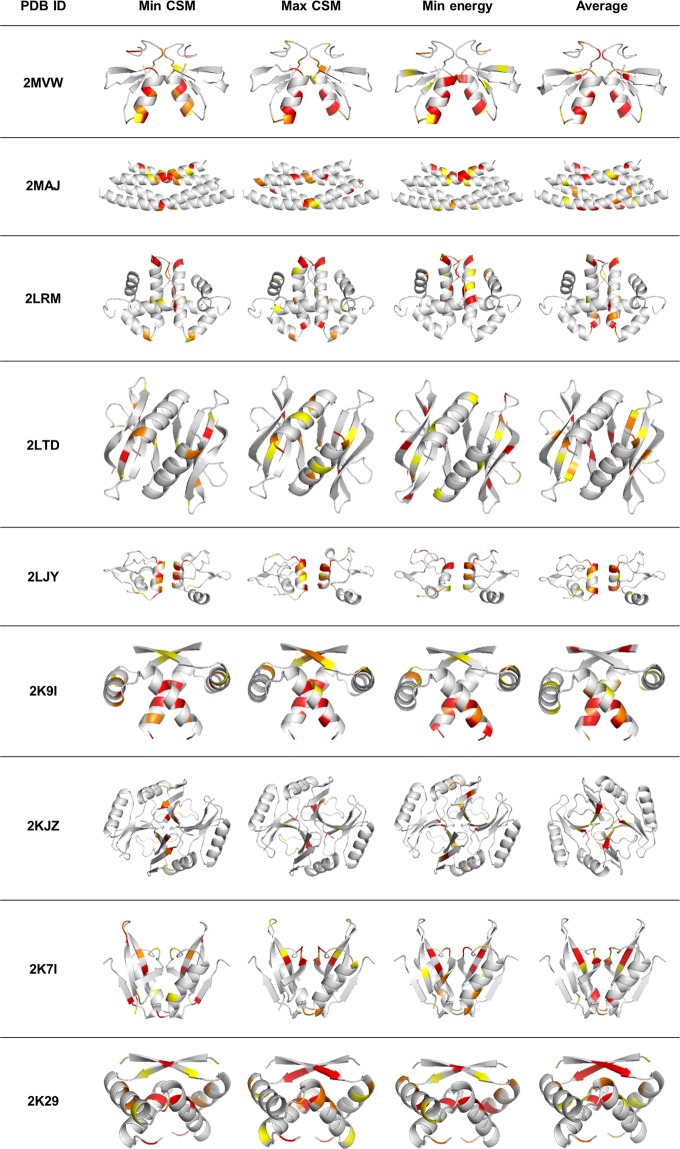
Symmetry maps of the conformers with the minimal CSM value, the maximal CSM and the minimal energy. The average conformer is discussed in the final section. The 10 most symmetry distorted symmetry-matched pairs of amino-acids are indicated. Color-code of the amino-acids: red - the 4 most symmetry-distorted pairs; orange - the next 3 most symmetry-distorted pairs; yellow - the next 3 most symmetry-distorted pairs.

Having the detailed analysis of the of the HPV16 E6 dimer at hand, we now turn to the full library of 41 oligomeric dimers, in order to see which of the identified ***C***_***2***_-symmetry trends of the conformers of HPV16 E6 characterizes this family of dimers in general. Figure 4 displays the symmetry maps of the three types of conformers analyzed above (minimal energy, most symmetric and least symmetric) for several dimeric proteins, and it is clear that the conclusion reached above, that the main burden of symmetry deviation lies in the touching zone of the two monomeric units for all conformers where the near-symmetry ***C***_***2***_ axis passes, is always the case. Table 2 (of all ***C***_***2***_-proteins which are mentioned in the main text) and Supplementary Table S1, (all other ***C***_***2***_-proteins), collect for each protein the ranges of the CSM values of all of its conformers (it also contains the CSM values of the averaged structures and of their B-factors to be explained and discussed in the last section), and Supplementary Fig. S3, collects representative examples of the CSM values distribution of the conformers. As in the detailed case we analyzed, it is seen that the CSM values of the conformers span typically over one order of magnitude. In particular we see that *none of the conformers in all proteins was found to be of exact C*_2_*-symmetry*. The imperfect symmetry of proteins oligomers seems to be a very general phenomenon; actually this is the rule and not the exception.

**Figure 5:**
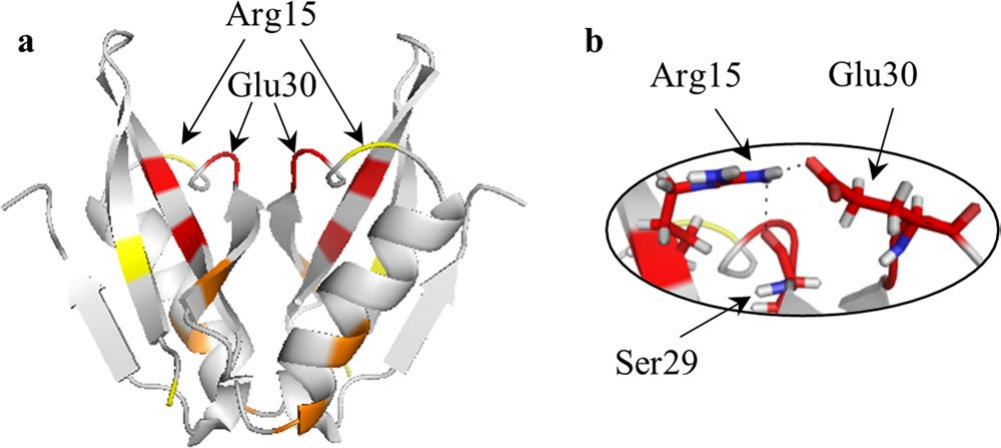
(**a**) Symmetry map of the NMR-derived minimal energy conformer of the ATU0232 protein (PDB 2K7I), and (**b**) a closer look at the asymmetric hydrogen bonds between Arg15 from one subunit and Glu30 from other subunit. Also shown is the hydrogen bond to Ser29 (black dashed lines).

It is not only that the overall picture of the dimeric proteins follows our opening detailed analysis of the HPV16 E6 dimer, but also the specific structural features of each of them follow similar details. For sake of brevity we shall briefly demonstrate it with one additional protein from the full list, namely ATU0232 from the Agrobacterium Tumefaciens (PDB 2K7I). We focus again on the three conformers - the minimal energy conformer, the least symmetry-distorted and the most distorted one – for which we identified the ten most-distorted-amino-acids pairs in each of them (see Supplementary Table S4), and built the resulting symmetry maps (Fig. 4, 2K7I). Interestingly, in this case the three conformers share only two amino-acids in the most distorted list (see Supplementary Table S4) - Glu30 and Arg15 - and these are indicated by arrows in Fig. 5a. A closer look at these amino acids (Fig. 5b) shows that the Arg15 from one subunit (the left one) forms two hydrogen bonds, one with Glu30 on the other subunit (the right one) and also with Ser29 from the left subunit. Because of this energy-optimal mutual orientation of Arg15 and Glu30, it is impossible to build a symmetry-equivalent hydrogen bond. This specific observation is another example that in the contact zone of the two units, each subunit usually adopts different conformations which lead to an optimal interaction, and which are expressed in the high CSM values.

We also used the symmetry maps and the local symmetry analysis in order to understand what characterizes the most symmetry-distorted pairs of amino-acids, besides their location in the protein structure, and in particular whether the hydrophilicity/hydrophobicity ratio of the amino-acids plays a role. Table 3 indicates that in most cases, the hydrophilic amino acids govern the distortion. This observation mirrors a similar conclusion reached by analysis of crystallographic data^13^. It is also seen that there is no significant change in the ratio of hydrophilic to hydrophobic amino-acids between the three conformers of each protein. It is not so trivial that even in the presence of water, still the main source of structure readjustment is due to multiple polar asymmetric interactions of hydrogen-bonds, and that water solvation does not interfere with that trend.

**Table 3:** The hydrophilic/hydrophobic ratio of the 10-most symmetry distorted amino-acids in several proteins.

PDB ID	Hydrophilic/hydrophobic ratio		
	Min enegy	Max CSM	Min CSM
2LTD	10:0	9:1	10:0
2K7I	9:1	8:2	9:1
2LJY	8:2	8:2	6:4
2MVW	8:2	6:4	6:4
2MAJ	7:3	8:2	8:2
2K9I	7:3	7:3	6:4
2K29	7:3	6:4	7:3
2KJZ	6:4	7:3	6:4
2LRM	4:6	6:4	7:3

Last but not least, it would be of interest to compare the same protein as determined by NMR and by crystallography. Surprising as it may appear, we were not able to locate such a study which fulfils the criterion of not assuming symmetry in the data analysis and which fulfils quality criteria listed in Methods. However, we were able to find a protein which was analyzed by the two methods in separate publications, and the comparison confirms all of the above conclusions: The protein is the homodimeric repressor protein CylR2 from *E. coli*, the structure was analyzed by NMR in reference^41^, and by crystallography in references^42,43^. The results of the comparative analysis are collected in Table 4, and again we see that the CSM values of the NMR-derived structures are one order of magnitude higher (0.11–0.20) than the CSM values of the two x-ray structures (0.015, 0.016). The local symmetry analysis of the three structures reveals that they share 4 pairs of amino acids (underlined) from the list of the 10-most distorted amino acids of each structure. Three of them (54, 57, 58) are, as expected, in the contact zone between the two subunits, and form hydrogen bonds between the components. These bonds, as repeatedly mentioned throughout the paper, affect the symmetry distortion, as carrying major part of the burden of getting away from ideal symmetry.

**Table 4:** A comparison of the symmetry deviations of NMR-derived structure to two crystalline states of homodimeric repressor protein CylR2 from *E.coli*.

PDB ID	Method	CSM value	10 most distorted amino acids
2LYJ	NMR	0.11–0.20	Ile3, Lys7, Lys13, Ser27, Ile34,Ser42, Asn54, Pro56, Leu57,Glu58
1UTX	x-ray	0.015	Ile3, Glu19, Ser27, Arg28, Gln29, Asn37, Asn40, Asn54, Leu57, Glu58
2XI8	x-ray	0.016	Met1, Ile3, Arg28, Gln29, Asn54, Pro56, Leu57,Glu58, Gln62, Glu66

The three structures share 5 pairs of amino acids (underlined) from the list of the 10-most distorted amino acids of each structure. All three have the same sequence. While 2LYJ and 2XI8 were treated with NaCl, 1UTX was treated with NaI.

### Domain swapping as a mechanism of symmetry distortion

**Figure 6:**
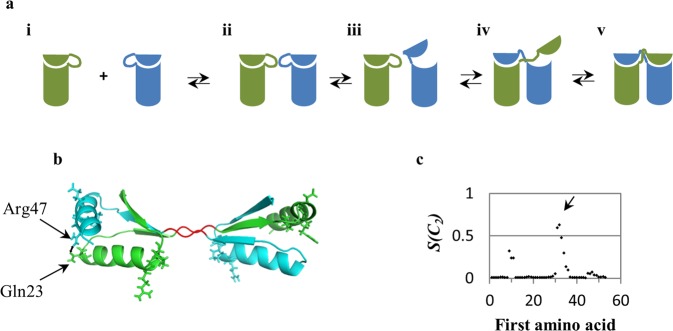
(**a**) The domain swapping mechanism, demonstrated on the formation of a dimeric oligomer: Two monomers with their folded hinge regions (i) encounter each other and form reversibly a transient dimer (ii). During the lifetime of (ii) a hinge of one monomer opens (iii) and its subunit replaces the equivalent subunit in the second monomer forming (iv), which then leads to the final swapped dimer (v). (**b**) The cytoplasmic domain of the *Escherichia coli* GlpG rhomboid protease (2MJA^44^). The two hinges are located at the center and are shown is red. Blue and green indicated the other ***C***_***2***_-symmetry related domains. The formed hydrogen bond between Arg47 and Gln23 is indicated by the two black arrows. (**c**) The continuous symmetry measure spectrum of the protein, in which the CSM value (*S(****C***_***2***_*)*) of the i-th segment (y-axis) is presented as a function of the position, *n*_*i*_, of the first amino acid in that specific segment (x-axis). The arrow indicates the hinge region. The two additional distortion peaks (at 46–48 and 9–11) - see text for their location and the origin of their distortion.

Symmetry distortion may provide also a clue on the mechanism of formation of the oligomer. In a previous study^14^ we have explored this possibility by focusing on the domain swapping mechanism of dimerization. By this mechanism, two monomeric units swap identical portions, resulting in an interwoven dimer as shown in Fig. 6a. We analyzed various crystallographic structures of proteins formed by this mechanism^14^, and found, using the CSM analysis, that the main burden of their symmetry distortion lies in this case in the hinge regions that connect the swapped portions. Furthermore, we showed that the CSM analysis clearly identifies the hinge region of swapped domain proteins, which has been considered to be a non-trivial task. Here we show that NMR structural data is of use for the detection and evaluation of this mechanism as well, and we extend our previous argument of why this mechanism must lead to symmetry distortion. Among the NMR structures that were analyzed in this study, the cytoplasmic domain of the *Escherichia coli* GlpG rhomboid protease (PDB 2MJA^44^) undergoes dimerization via domain swapping mechanism (Fig. 6b), as suggested by Ghasriani *et al*.^44^. The method for detection the most distorted region for this mechanism is the CSM spectrum analysis, described in Methods. We have carried out the analysis on this protein structure; the resulted symmetry spectrum (Fig. 6c) indicates a sharp peak at amino-acids positions range of 32–34. This segment very closely coincides, with minor shift of one residue, with the originally suggested hinge region at the amino-acids 33–35, shown in red in Fig. 6b. This is the most symmetry-distorted segment in the protein: it carries the main burden of the overall symmetry distortion of the structure. Additional distorted regions are indicated in the spectrum, at the 9–11 and 46–48 segments (and their symmetry-related counterparts in the second subunit of the dimer). Examination of the 3D structure (Fig. 6b) shows the origin of the 46–48 distortion. The segment 46–48 in one subunit forms a hydrogen bond between Arg47 and Gln23 from the other subunit (Fig. 6b). However, the symmetry related amino acids do not form this hydrogen bond, probably because of the “scissors-like” closing of one side which causes opening on the other side of the Arg47 and Gln23 neighboring amino acids. The origin of the symmetry distortion of the 9–11 segment (Fig. 6b, marked by sticks representation) is not clear, but probably represents the combined effects of the hinge and the 46–48 segment distortions. We propose that the swap mechanism must lead to distortion because it is very likely a sequential process and not a concerted one, as detailed in Fig. 6a.

### Higher symmetry oligomers

**Figure 7:**
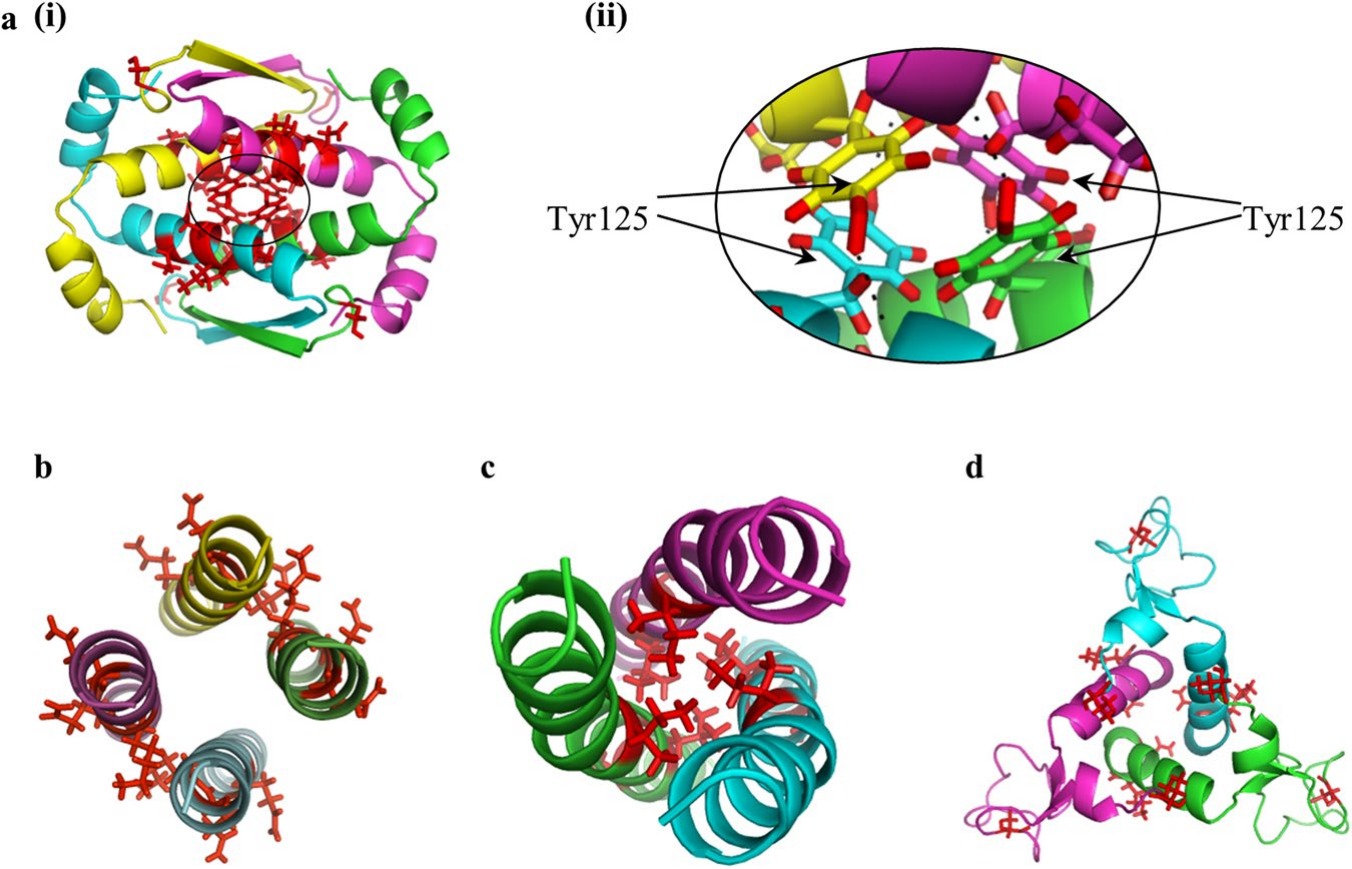
The symmetry distortions in ***D***_***2***_ and ***C***_***3***_ proteins (each subunit is in a different color) - the most distorted amino acids in the most- and least-symmetry-distorted conformers are colored red. ***D***_***2***_-symmetry: **(a)** Ciona intestinalis p53/p73 protein (PDB 2MW4, CSM range: 0.054–0.13), and (**b**) heterogeneous nuclear ribonucleoprotein (hnRNP) C (PDB 1TXP, CSM range: 0.34–0.66). ***C***_***3***_-symmetry: (**c**) The oligomerization domain of cartilage matrix protein (PDB 1AQ5, CSM range: 0.014–0.055) and (**d**) anti-TRAP trimer (PDB 2KO8, CSM range: 0.04–0.28).

There is significantly less available structural NMR data on oligomers of higher symmetries, but such reports – particularly with ***C***_***3***_ and ***D***_***2***_ – do exist. In these higher symmetry point groups the geometrical possibilities of assembly into oligomers are wider, and as seen in Fig. 7, they may adopt structures, such as a triangle, in which the zones of interaction between the monomeric units are less straightforward compared to what we have seen in the dimers. However, in these contact zones, the general trend identified so far, persists: the most distorted amino acids are located in the interacting zone of the oligomer subunits. Let us take for example the tetramerization domain of the ***D***_***2***_-symmetry-protein, Ciona intestinalis p53/p73 protein (pdb 2MW4^45^), Fig. 7a(i). Here there is an extensive contact area between the subunits and the optimal interaction between them requires the components to adjust to each other. Two conformers of the protein were analyzed in detail, the most- and least-symmetry-distorted conformers. For each one of them, the 10 most symmetry distorted pairs of amino acids were determined; we found that these two conformers share 6 quadruples of distorted amino-acids, out of the 10 – see Supplementary Table S5. As can be seen in Fig. 7a(i), most of them are located in the contact zone of the subunits, and a closer look at the interface, Fig. 7a(ii), shows that 4 hydrogen bonds between the hydroxyl group of Tyr125 and the backbone of other subunit are formed. That is, each of the four subunits in the oligomer acquires a different conformer, which is mainly different in the central touching interface.

As mentioned above, high symmetries can pack the monomer units with limited contacts, and the ***D***_***2***_-symmetric heterogeneous nuclear ribonucleoprotein (hnRNP) C protein (pdb 1TXP^46^) is an example for such a case (Fig. 7b): The main contact is at the interface between the monomers of each of the two dimers, but not between the pair of dimers. The ***C***_***3***_ proteins - Fig. 7c,d, contain three interfaces between the three monomeric units, and it is interesting to check if their distortions are identical or different: Identical distortions may hint to a concerted trimerization, while different distortion may point to a sequential process, by the same argument we have used for the swap mechanism.

### What can solution NMR conformers tell us about the crystallographic structure?

As already mentioned in the Introduction, X-ray crystallography and NMR spectroscopy provide structural information of proteins relevant to different environments – the crystalline state and the water-dissolved state^47^. However, it has been shown that structure determination based on x-ray or NMR (not necessarily symmetric oligomeric proteins) usually shows similar conformation of the main chain but differ in details concerning the packing of surface loops and side chains^47,48^. One of the most significant differences between the methods is the availability of information regarding the dynamics or macromolecular motion of the protein^49^. Whereas X-ray diffraction experiment provides a spatial and a temporal average, NMR experiments encompass many important motions of the proteins^47^. Here we wish to highlight another possible connection between these two types of analyses:

The fact that NMR analysis identifies a family of conformers in solution raises an interesting question – when a protein crystallizes from solution, which - if at all - of the conformers is used for the specific building the repetitive, translational symmetric crystal? In the following discussion we raise the conjecture that a crystal of a protein is not classical, from the point of view that the crystal is built from various conformers, and that the picture of exact translational symmetry of a unit cell would better be replaced by a picture of a collection of similar – not identical – unit cells. These different unit cells are either randomly distributed or clustered in small domains within the crystal. As mentioned above, we are not the first to think in this direction. For instance, it has been suggested by Woldeyes *et al*.^20^ that in the crystal lattice, protein molecules can adopt multiple different conformers. In particular they proposed that the crystallographic structure which is produced is an average of the conformational ensemble in time and space, and they wonder about the relation of the B-factor (the thermal factor) to this phenomenon. Here is our analysis of this proposed phenomenon, which emerges from the symmetry analyses presented above, relating also to the B factor. To do so we first create for a given oligomer an averaged NMR-derived structure, which is based on the coordinates of all its conformers. This is carried out by averaging the coordinates of each and the same atom in each of the conformers. Typical averaged structures are shown in Fig. 4 right column, and it is seen that, importantly, the identified distortive features described above, are retained. However, there is a major difference between these averaged structures and the other three conformers shown in each row - we recall that the color code refers to the ten most distorted amino-acid pairs, but if the actual CSM values are looked at, they are found to be much lower for the averaged conformer, that is, it is much more symmetric. In fact, if that conformer would have been drawn on the color code of one of the conformers, it would appear almost fully gray. Indeed, it can be seen in Table 2 that the overall CSM values of the averaged conformers are by far lower than even the most symmetric conformer, typically more than one order of magnitude lower, a gap which is even much higher for the most distorted conformer. The average geometry is expected to be the much more symmetric than each of the conformers, because the directionalities of the conformational changes - vectorial entities that can have negative values - tend to compensate each other. The striking observation is that the low CSM values of the averaged conformers are similar in their magnitude to those obtained from the analysis of the crystallographic structures, as reported previously: Typical CSM values from x-ray data analyses^13^ are of the order 10^–3^, of the same order of magnitude as the averaged conformers structures.

To see if this similarity goes beyond the CSM values, we also calculated the apparent B-factor for each atom of the average structures, based on the family of the conformers as follows: First we calculated U, the mean square displacement, for each atom and for each of the three Cartesian axes:

2 $${U}_{{x}_{atom}}=\sqrt{\frac{\sum {({x}_{i}-\bar{x})}^{2}}{n}},$$

where $n$ is the number of the conformers in the NMR ensemble, $i$ is the conformer index ($i=1,\,2,\ldots ,\,N$), ${x}_{i}$ and $\bar{x}$ are the atom coordinate of the *i*^th^ and the averaged conformers, respectively. This is repeated for the two additional axes, obtaining ${U}_{y}$ and ${U}_{z}$. We then calculated the B-factor for each atom and for each axis:

3 $${B}_{{x}_{atom}}=8{\pi }^{2}{U}_{{x}_{atom}}^{2},$$

obtaining similarly ${B}_{{y}_{atom}}\,{\rm{and}}\,{B}_{{z}_{atom}}$, and then calculated the effective B-factor for each atom:

4 $${B}_{atom}=\frac{{B}_{x}+{B}_{y}+{B}_{z}}{3},$$

Finally we calculated the average B-factor of all atoms of a given protein from:

5 $${\bar{B}}_{factor}=\frac{{\sum }^{}{B}_{i}}{N}$$

where *N* is the number of atoms in the protein, and *i* is the atom index ($i=1,\,2,\ldots ,\,N$).

The resulting B factors are collected in Table 2, right column. Again, strikingly, the range of the effective average B-factor for all calculated proteins is the same order as of x-ray structures^50^.

We propose that the similarities of the of the CSM values of the averaged NMR structure and of their apparent B-factors to those observed in crystallography, is more than a coincidence, and that it points to the possibility that what one sees in the analysis of proteins x-rays data are crystals composed of several conformers. Crystallization of proteins from water may start from more than one semi-stable conformer, and the continuous growth then can proceed with any other conformer that co-exists in solution. Furthermore, by the same token that we saw the interface of the monomeric units in an oligomer adjusting to each other at the point of contact, lowering the oligomeric symmetry, such adjustment may occur at the touching zone of one unit-cell in the crystal which occupies a certain conformer, to the next unit cell which is occupied by a similar, not necessarily the same conformer; this will further lower the translational symmetry of the crystal. We are not certain yet, what experimental method can test this hypothesis.

## Conclusions

We provided a quantitative tool to evaluate the degree of symmetry distortion of protein homomer, based on solution NMR-derived structural information. As this methodology provides a family of conformers for each protein, the full families were analyzed, and their symmetry properties compared. We found that as a rule, the distortion is mainly due to hydrophilic amino acids at the contact zone of the monomeric units. While departure from ideal symmetry may be favored for enthalpic reasons increasing the energy release when two monomers interact, one should also consider the entropy gain in giving away perfect symmetry: as the protein deviates from its perfect symmetric structure, that number of possible microscopic conformations increases sharply and the entropy content rises. Compared to the symmetry distortions found in the crystalline form from the analysis of x-ray data, symmetry deviations in solution are much higher – by about one order of magnitude based on the Continuous Symmetry Measures methodology – compared to solution. One of the main parameters that leads to the deviation from exact symmetry is the process of forming the homomer, and in that context we analyzed from the point of view of symmetry the domain swap mechanism, and identified the hinges formation as the main source of the symmetry deviation. Our analysis leads to the proposition that the lower symmetry deviations detected in crystals reflect in fact the averaging contributions of several conformers that take part in building the same crystal.

## Supplementary information

Supplementary information.

## Data Availability

The data that support the findings of this study are available from the corresponding author upon reasonable request.
