# Supplementary material for: The near-symmetry of protein oligomers: NMR-derived structures
Source: Sci Rep. 2020 May 20;10:8367. doi: 10.1038/s41598-020-65097-8 (PMC7239866; doi:10.1038/s41598-020-65097-8)
Supplement: Supplementary file 1 — Supplementary information. [file 41598_2020_65097_MOESM1_ESM.pdf]

The near-symmetry of protein oligomers: NMR-derived structures

Maayan Bonjack and David Avnir*

Institute of Chemistry, The Hebrew University of Jerusalem, Jerusalem 9190401, Israel

* Corresponding author
E-mail: david.avnir@mail.huji.ac.il

Supplementary Material

Table S1. Additional entries to Table 2: The CSM values of the most and least distorted conformers and of the averaged structures. The average effective B-factor for each protein structure, based on the displacement of the atomic positions in NMR conformers from the averaged artificial conformer is also shown and is discussed in the final section of the paper.

PDB ID	Min CSM	Max CSM	Averaged structure CSM	Average B-factor
2MMV	0.0891	0.3071	0.0195	40.27
2MRN	0.0031	0.0077	0.0007	26.19
2MF2	0.0014	0.0089	0.0004	60.85
2MAB	0.0250	0.0975	0.0079	7.74
2MHE	0.0982	0.2235	0.0040	31.33
2LZF	0.1299	0.4109	0.0194	43.54
2LW9	0.4807	0.8137	0.1953	51.59
4AAI	0.2428	0.7114	0.1435	27.06
2KQM	0.0464	0.1446	0.0041	22.66
3ZTG	0.0052	0.0453	0.0012	7.14
2L48	0.0358	0.1065	0.0112	19.21
2KOD	0.0016	0.0122	0.0019	25.16
2KO1	0.1176	0.2033	0.0112	21.85
2K01	0.0932	0.3018	0.0142	44.38

2JRL	0.0072	0.0502	0.0017	27.70
2JWK	0.0025	0.0050	0.0109	14.67
2JZ0	0.0663	0.1899	0.0102	12.81
2RMM	0.0671	0.2429	0.0172	18.95
2DO6	0.0575	0.1804	0.0089	19.47
2GJF	0.0684	0.3776	0.0076	18.81
2FI2	0.0494	0.2003	0.0067	32.40
2B95	0.1652	0.3114	0.0149	30.76
1ZAE	0.1331	0.7419	0.0132	41.01
1YSF	0.0069	0.0141	0.0039	21.33
1Q6B	0.0006	0.0132	0.0009	25.20
1I18	0.0025	0.0051	0.0008	15.93
1WJB	0.0063	0.0247	0.0040	19.51

Figure S1. Comparative symmetry maps of three NMR-derived conformers of the N-terminal domain dimer of HPV16 E6 (PDB 2LJY): (**a**) The conformer with the minimal energy. (**b**) The least $\boldsymbol{C_2}$-symmetry distorted conformer. (**c**) The most symmetry distorted conformer. Note that because of the near $\boldsymbol{C_2}$-symmetry, the left arm colors of the dimer are in the back of the right arm, and *vice-versa*. Only the 10-most distorted pairs of amino acids are colored. Here, for all conformers, red 1.0 < S($\boldsymbol{C_2}$); orange 0.5 < S($\boldsymbol{C_2}$); yellow S($\boldsymbol{C_2}$) < 0.5); grey – the rest of the amino acids. See Table 1 for details.

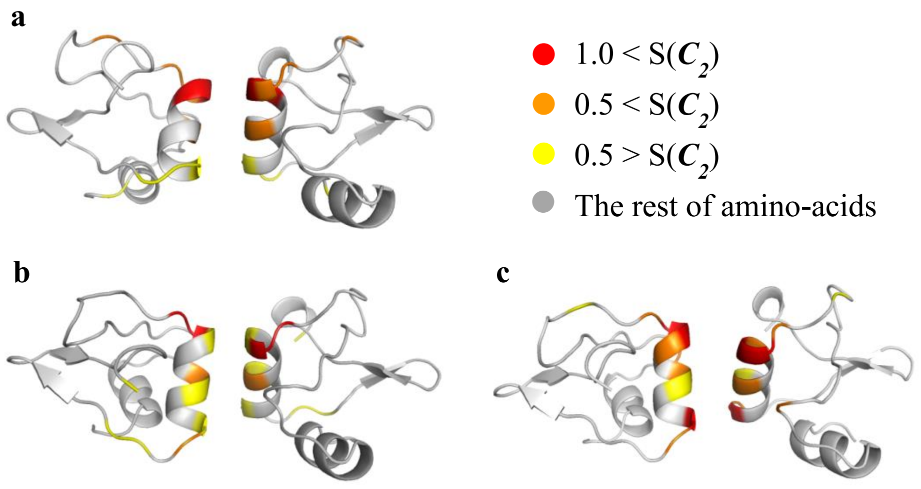

Figure S2. (**a**) Residues interactions across interface of the N-terminal domain dimer of HPV16 E6 (PDB 2LJY) (based on "PDBsum: a web-based database of summaries and analyses of all PDB structures", which are colored red in (**b**). The contribution of the interface residues is of 0.44 out of the protein overall CSM value of 1.39.

a

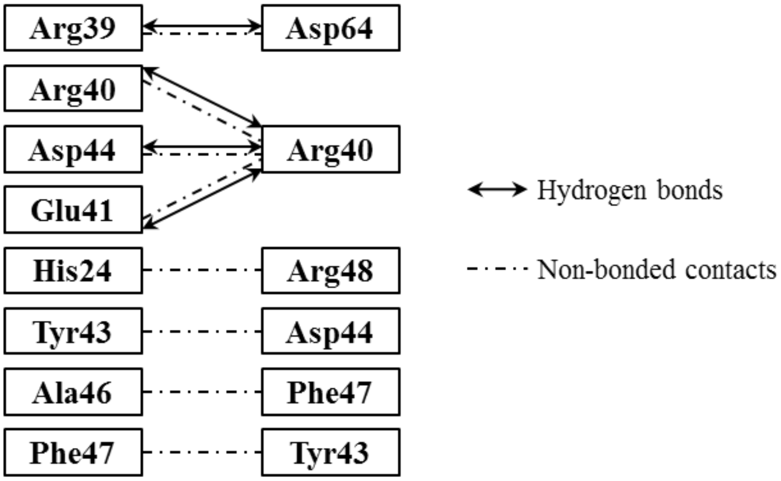

b

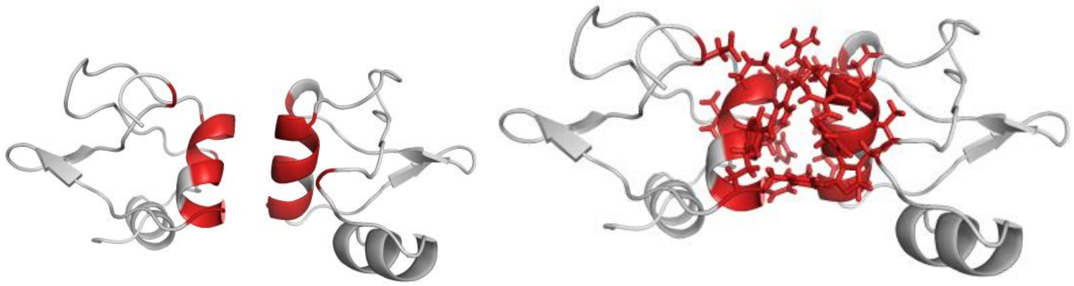

Table S2. Comparison of the local symmetry analyses of three conformers of the N-terminal domain dimer of HPV16 E6 (pdb code: 2LJY) calculated by different methods: The local CSM value for each pair of amino acids based on the best $\mathbf{C_2}$ axis determined just for the pair of residues (Best local $\mathbf{C_2}$ axis) compared with that based on the axis obtained for the whole protein dimer (Best global $\mathbf{C_2}$ axis).

Minimal energy

Best local $\mathbf{C_2}$ axis		Best global $\mathbf{C_2}$ axis	
Residue number	S($\mathbf{C_2}$)	Residue number	S($\mathbf{C_2}$)
40	19	40	22
41	2.1	41	2.1
38	1.0	38	1.1
35	0.77	43	0.83
43	0.59	35	0.78
39	0.58	49	0.59
49	0.499	39	0.59
47	0.44	48	0.55
48	0.41	64	0.54
11	0.38	46	0.54

Least distorted

Best local $\mathbf{C_2}$ axis		Best global $\mathbf{C_2}$ axis	
Residue number	S($\mathbf{C_2}$)	Residue number	S($\mathbf{C_2}$)
38	1.8	39	1.8
39	1.3	38	1.8
43	0.71	43	0.71
48	0.48	48	0.54
47	0.37	47	0.37
44	0.24	44	0.33
51	0.21	67	0.24
67	0.20	51	0.21
50	0.19	50	0.21
40	0.16	40	0.16

Most distorted

Best local $\mathbf{C_2}$ axis		Best global $\mathbf{C_2}$ axis	
Residue number	S($\mathbf{C_2}$)	Residue number	S($\mathbf{C_2}$)
40	14	40	23
39	4.8	47	9.1
47	2.3	39	4.9
38	1.0	43	2.8
41	0.91	38	1.2
48	0.86	41	1.0
43	0.83	48	0.99
24	0.80	24	0.89
35	0.46	35	0.46
44	0.37	44	0.37

Figure S3. Representative conformer CSM distribution graphs of proteins from tables 2 and S2: (a) 2LJY, (b) 2MVW, (c) 2MX9, (d) 2MFZ, (e) 2MGS, (f) 2LYJ.

a

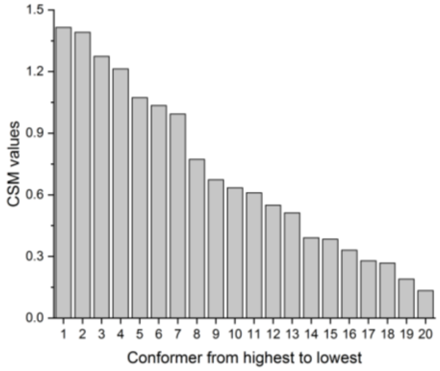

b

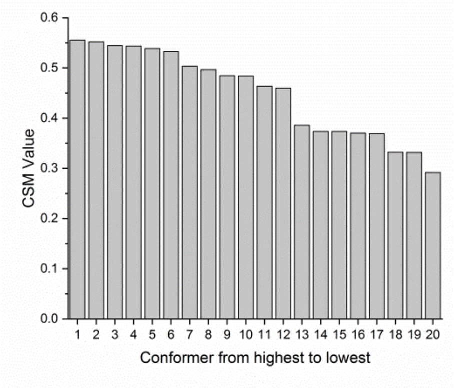

c

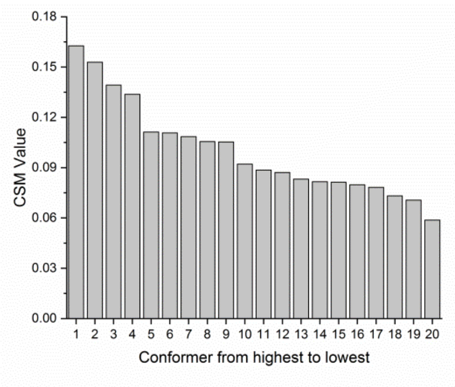

d

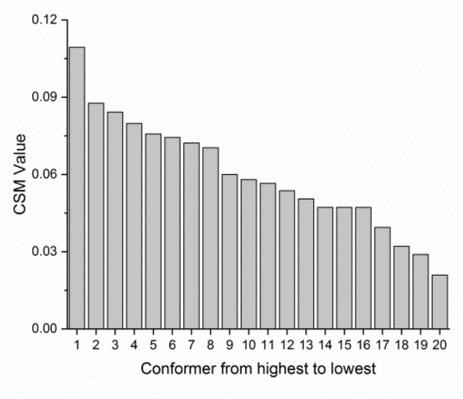

e

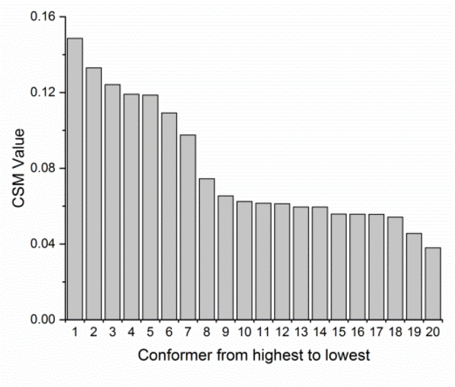

f

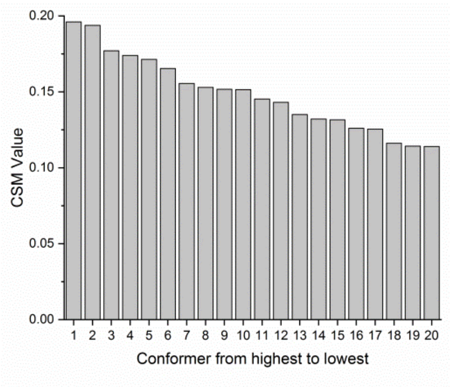

Table S3. CSM values of the 20 conformers of the N-terminal domain dimer of HPV16 E6 (pdb code: 2LJY) with and without the hydrogen atoms.

**CSM values of conformers**	
With hydrogens atoms	Without hydrogens atoms
1.39	1.54
0.67	0.60
0.38	0.26
0.19	0.16
0.55	0.56
0.14	0.09
0.27	0.29
0.63	0.49
1.07	1.20
0.39	0.38
0.99	0.77
1.21	1.32
0.33	0.31
1.03	0.95
0.61	0.43
0.51	0.50
1.27	1.62
0.77	0.82
1.42	1.58
0.28	0.27

Table S4. The ten most distorted amino acid residues in the three conformers of the ATU0232 protein (Fig. 5 – 2K7I) and their local S($\mathbf{C_2}$) symmetry distortion CSM values.

**Minimal energy** (residue number [S($\mathbf{C_2}$)])	**Least distorted** (residue number [S($\mathbf{C_2}$)])	**Most distorted** (residue number [S($\mathbf{C_2}$)])
1 [0.33]	1 [0.24]	
5 [0.38]		
	9 [0.49]	
	10 [0.32]	10 [0.23]
15 [1.5]	15 [0.28]	15 [0.64]
		16 [12]
17 [0.60]	17 [1.0]	
	22 [0.25]	
24 [0.42]		
		27 [2.9]
		28 [1.1]
29 [3.1]		29 [5.5]
30 [3.0]	30 [0.10]	30 [5.3]
33 [0.34]		
		35 [0.63]
43 [0.43]		
	44 [0.11]	
47 [0.40]		47 [0.95]
	48 [1.2]	48 [1.4]
	53 [0.44]	

Table S5. The ten most distorted amino acid residues in the three conformers of Ciona intestinalis p53/p73 protein (Fig. 7a – 2MW4) and their local S($\boldsymbol{C_2}$) CSM values.

**Minimal energy** (residue number [S($\mathbf{C_2}$)])	**Least distorted** (residue number [S($\mathbf{C_2}$)])	**Most distorted** (residue number [S($\mathbf{C_2}$)])
103 [0.27]	103 [0.16]	103 [0.29]
		104 [0.24]
		114 [0.14]
	117 [0.053]	117 [0.16]
122 [4.9]	122 [0.11]	122 [4.9]
123[0.42]	123 [0.41]	123 [0.60]
124 [0.73]	124 [0.49]	124 [0.76]
125 [0.082]	125 [0.10]	125 [0.17]
127 [0.38]	127 [0.10]	127 [0.40]
	133 [0.19]	
136 [0.19]	136 [0.14]	
140 [0.10]		140 [0.13]
146 [0.073]		
147 [0.11]	147 [0.076]	
